# Left ventricular systolic function changes in hypertrophic cardiomyopathy patients detected by the strain of different myocardium layers and longitudinal rotation

**DOI:** 10.1186/s12872-017-0651-x

**Published:** 2017-08-02

**Authors:** Jun Huang, Zi-Ning Yan, Li Fan, Yi-Fei Rui, Xiang-Ting Song

**Affiliations:** Department of Echocardiography, ChangZhou No.2 People’s Hospital Affiliated to NanJing Medical University, ChangZhou, 213003 China

**Keywords:** Left ventricular, Hypertrophic cardiomyopathy, Strain, Longitudinal rotation

## Abstract

**Background:**

Impairment of left ventricular (LV) longitudinal function has an important role in hypertrophic cardiomyopathy (HCM). This research investigated an association between the longitudinal strain of different myocardial layers, longitudinal rotation and the LV systolic function of HCM patients.

**Methods:**

The research was performed on 36 HCM patients and 36 healthy subjects. The peak systolic longitudinal strain of the subendocardial, midmyocardial, and subepicardial layers was measured using 2-dimensional speckle tracking echocardiography (2D–STE). The apical long-axis and 4- and 2- chamber views were acquired via 2D Doppler echocardiography. The curve of the longitudinal rotation was traced at 17 timepoints in the analysis of 2 cardiac cycles.

**Results:**

Compared with healthy subjects, in HCM patients regional LV peak systolic longitudinal strain was less, not only in hypertrophied LV myocardium, but also in non-hypertrophied myocardium. The rotational degrees of the midmyocardial-septal, apex, and lateral wall of HCM patients were significantly different from that of normal subjects, as follows. In HCM patients, clockwise longitudinal rotation was found. The interventricular septum thickness at end-diastole positively correlated with the peak longitudinal systolic strain of the subendocardial, the midmyocardial, and the subepicardial layers. The area under ROC curve values for subendocardial, midmyocardial and subepicardial layers in HCM patients were 0.923, 0.938, 0.948.

**Conclusion:**

In HCM patients, the longitudinal function was damaged, even with normal LV ejection fraction. The peak longitudinal systolic strain of the subendocardial, midmyocardial, and subepicardial layers, and the longitudinal rotation detected by 2D–STE, are very sensitive predictors of systolic function in patients with HCM.

## Background

Hypertrophic cardiomyopathy (HCM) is a common clinical heart disease. It is a genetic disorder of the myocardium caused by mutations in cardiac sarcomeric proteins, with asymmetric hypertrophy of the left ventricle, right ventricle, or both [1–3]. The interventricular septum is always involved. Based on the degree of left ventricular (LV) outflow tract obstruction, HCM can be categorized at rest as nonobstructive (no obstruction or provocation), labile, or obstructive, with peak gradients <30 mmHg, >30 mmHg only during provocation, and >30 mmHg, respectively [4]. Most patients with HCM present with no clinical symptoms, but signs are often found during echocardiography, computed tomography scan, or magnetic resonance imaging [5–7]. Some patients may suffer sudden cardiac death due to ventricular tachycardia or fibrillation [8].

With the development of various imaging techniques, especially echocardiography, the discovery and diagnosis of HCM by conventional 2-dimensional ultrasound is becoming more easy and convenient. While the LV ejection fraction (LVEF) can sometimes reflect the systolic function of the left ventricle, it is not reliable, as the LVEF of most HCM patients is normal. Tissue Doppler imaging detects velocity and strain and is one of the most used echocardiography methods [9, 10], but angle dependency is not reproducible [11]. Two-dimensional (2D) speckle tracking echocardiography (STE) is a new technique that tracks frame-to-frame movement of natural acoustic markers. This enables the measurement of velocity, strain, strain rate, and torsion so that the ventricular or atrium function can be assessed [12–16]. While 2D–STE is angle-independent, out-of-plane motion often makes the results not particularly accurate [17]. Three-dimensional (3D)-STE can be used to assess LV function [18, 19], but the frame rate prevents accuracy, and therefore 3D–STE depends on the quality of 2D images for acquisition and suffers in lower temporal and spatial resolution [20].

To evaluate the changes in LV longitudinal systolic function in HCM patients, the following are innovations of the present study: The anatomy of normal myocardium consists of subendocardial, middle wall and subepicardial myocardial fibers, Using multilayer strain to analysis the LV function is a new method, so the first aim is to measure the peak systolic longitudinal strain of the subendocardial, midmyocardial, and subepicardial layers in patients with HCM; Longitudinal rotation as a new marker has received little attention. The LR means the rotational motion in the long axis of heart, but the origin of LR is still unclear, so the second aim is to Phase a hypothesis that there was longitudinal rotation of the cardiac in HCM patients, measure the longitudinal rotation in HCM patients; and tracing the curve of the LV longitudinal rotation motion in HCM patients by 2D–STE, and then to verify the hypothesis. Last to assess the changes in LV longitudinal systolic function in HCM patients.

## Methods

### Ethical approval

The Human Subjects Committee of Changzhou No. 2 People’s Hospital approved this study. Written informed consent was obtained from the each couple enrolled in the study.

### Study sample

Thirty-six HCM patients and 36 age-and gender-matched healthy (normal control) subjects were enrolled. The diagnosis of HCM was based on the following M-mode and 2D echocardiographic evidence of wall thickness ≥ 15 mm in one or more LV myocardial segments and non-dilated left ventricle (LV). In addition to the absence of another cardiac or systemic disease capable of producing the magnitude of hypertrophy evident in patients with HCM, such as the valve diseases valve stenosis, hypertensive heart diseases, and coronary heart disease. Apical HCM patients were excluded for the study. If the ECG showed LBBB, HCM patients were excluded for the study. All enrolled HCM patients were non-obstructive, based on the degree of LV outflow tract obstruction, there was no obstruction at rest or provocation (peak gradient <30 mmHg). All enrolled HCM patients were had septal wall hypertrophy and with/without other LV walls hypertrophy.

The normal control subjects had no evidence or family history of HCM, hypertension, diabetes mellitus, or any other disease; all of the physical examination tests, the electrocardiogram, and the echocardiograph were normal. Recruitment to the study followed a full explanation of our methods, including that there was no risk of harm.

### Conventional 2D Doppler echocardiography

All 36 HCM patients and 36 normal subjects underwent conventional 2D Doppler echocardiography (Vivid E9, GE). Left atrial diameter, interventricular septum thickness at end-diastole (IVSD), and LV posterior wall thickness in end-diastole (LVPWD) were measured in the parasternal long axis view of the LV by M-mode. Simpson’s biplane method was used to measure the LVEF. The peak early and late diastolic mitral annular velocities (Ve and Va, respectively) were measured by pulsed-wave Doppler, and the ratio of early diastolic inflow-to-late diastolic flow at the mitral valve (Ve/Va) was calculated.

In each group, ECG leads were connected to each individual. For offline analysis, the following were acquired: hold on the breath, standard high frame rate (60–90/s) of the apical long-axis, and 4 and 2-chamber views of 3 consecutive cycles.

### Data analysis for LV systolic function

We analyzed the apical long-axis and 4- and 2- chamber views using 2D–STE software (2D–Strain, EchoPac PC v.7.x.x, GE Healthcare, Horten, Norway). Each of the LAX, A4C, and A2C options were used to sketch the LV subendocardial layer. The aortic valve closure time in the apical long-axis view was confirmed. The software then automatically created a region of interest (ROI) which contained the subendocardial, midmyocardial, and subepicardial layers. The ROI was adjusted to include the myocardial as well. In the ROI, the software divided the LV into 6 segments. The peak systolic longitudinal strain of the subendocardial, midmyocardial, and subepicardial layers were calculated.

We defined longitudinal rotation as the global rotation of the LV cross section. The subendocardium layer was displayed by using the SAX-MV of the Echopac in the apical 4-chamber view. The software automatically created a ROI that included the subendocardial, midmyocardial, and subepicardial layers. The ROI was adjusted to include the subendocardial and subepicardial layers. The LV region was divided into five segments: base-septal, midmyocardial-septal, apex, midmyocardial-lateral and base-lateral. The segmental longitudinal rotation of the LV was assessed in the same view via 2D–STE.

The longitudinal rotational degrees in the apical 4-chamber views were measured at 17 timepoints in the analysis of 2 cardiac cycles (each measured from onset-to-onset of the QRS wave): onset of QRS wave; mitral valve closure; mid-isovolumic contraction; aortic valve opening; 25%, 50%, and 75% of ejection phase; aortic valve closure; mid-isovolumic relaxation; mitral valve opening; peak early diastole and end of early diastole; onset, peak, and end of atrial filling; onset of the second QRS wave; and aortic valve opening of the second heart cycle [21–23].

The time between mitral valve closure and aortic valve opening was considered the isovolumic contraction. The time from aortic valve opening to aortic valve closure was considered the ejection period. The time between aortic valve closure and mitral valve opening was defined as isovolumic relaxation. The time from mitral valve opening to mitral valve closure was the diastole period.

### Statistical analysis

All of the analysis was performed using SPSS 17.0 software (SPSS, Chicago, IL, USA). Data are presented as the mean ± standard deviation. Any difference was considered statistically significant in all tests when the *P*-value was less than 0.05. To determine normality, the distribution of the peak longitudinal systolic strain of the subendocardial, midmyocardial, and subepicardial layers in all subjects was assessed using the Kolmogorov-Smirnov’s test. If the data distribution was normal, differences between the HCM patients and normal subjects were compared with an independent Student’s t-test. For variables with a non-normal distribution, the nonparametric Mann-Whitney test was used. The correlation between the IVSD and the peak longitudinal systolic strain of the subendocardial, midmyocardial, and subepicardial layers was determined by Pearson’s correlation if the data distribution was normal. For variables with a non-normal distribution, Spearman’s correlation was chosen. We defined the peak longitudinal systolic strain values of different layers in control subjects as the normal state, and considered the values of HCM patients as abnormal. The values for measuring the peak longitudinal systolic strain of subendocardial, midmyocardial and subepicardial in HCM patients were determined from receiver operating characteristic (ROC) curve analysis. Yoden’s index was selected for the cut-off point which can give the best composite of specificity and sensitivity.

## Results

### Basic information in HCM patients and the normal subjects

There were significant differences in left atrial diameter, IVSD, LVPWD (*P* < 0.01; Table 1). In HCM patients, the left atrial diameter, IVSD, and LVPWD were significantly larger than in the control subjects. Between the HCM and control subjects the following were statistically similar: LVEDV, LVESV, LVEF, Ve and Va and Ve / Va, (*P* > 0.05).

**Table 1:** Basic Information in HCM patients and normal subjects from conventional Two-Dimensional Doppler Echocardiography (mean ± s.d.)

	HCM (36)	Normal (36)	*P*-Value
Age(yrs)	47 ± 14	46 ± 12	0.703
Male gender(%)	64	61	
HR(bpm)	72 ± 12	73 ± 12	0.343
LAD(mm)	42 ± 5	35 ± 4	<0.001
IVSD(mm)	19 ± 4	9 ± 1	<0.001
LVPWD(mm)	10 ± 1	9 ± 1	<0.001
LVEDV(ml)	80 ± 18	84 ± 11	0.099
LVESV(ml)	27 ± 9	30 ± 8	0.333
LVEF(%)	67 ± 6	65 ± 6	0.087
Ve(m/s)	0.79 ± 0.26	0.85 ± 0.15	0.205
Va(m/s)	0.62 ± 0.23	0.69 ± 0.18	0.167
Ve/Va	1.45 ± 0.67	1.31 ± 0.36	0.259

*LAD* left atrial diameter, *HR* heart rate, *IVSD* interventricular septal thickness in end-diastolic period, *LVPWD* left ventricular posterior wall thickness in end-diastolic period, *LVEDV* left ventricular end-diastolic volume, *LVESV* left ventricular end-systolic volume, *LVEF* left ventricular ejection fraction, *Ve* the peak velocity during early diastole of anterior mitral leftlet, *Va* the peak velocity during late diastole of anterior mitral leftlet

### Peak systolic longitudinal strain in different myocardium layers

**Fig. 1:**
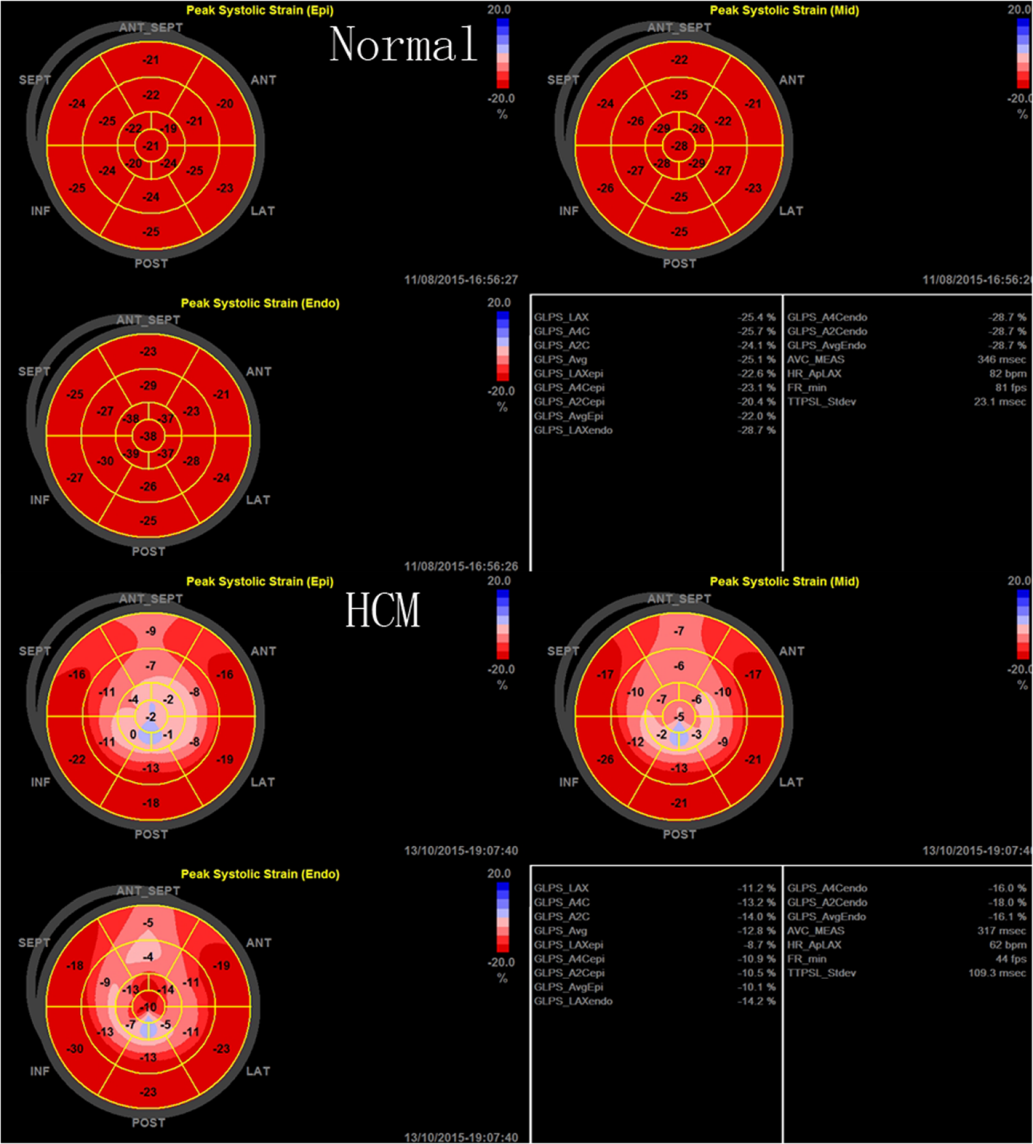
The bull’s eyes of the peak systolic longitudinal strain of the subendocardial, midmyocardial and subepicardial layers between normal subjects and HCM patients

The trend of the peak systolic longitudinal strain of the subendocardial, midmyocardial, and subepicardial layers in all the subjects was: subendocardial > midmyocardial > subepicardial (Table 2, Fig. 1). All systolic peak longitudinal strains were different between the HCM and normal subjects. In HCM patients, the LV peak systolic longitudinal strain was lower than in the normal subjects, not only in hypertrophied LV myocardium, but also in non-hypertrophied myocardium.

**Table 2:** Comparision of the peak systolic longitudinal strain of the subendocardial, midmyocardial and subepicardial layers in HCM patients and normal subjects (mean ± s.d.)

		Subendocardial			Midmocardial			Subepicardial		
		HCM(36) (%)	Normal(36) (%)	*P*-Value	HCM(36) (%)	Normal(36) (%)	*P*-Value	HCM(36) (%)	Normal(36) (%)	*P*-Value
3-CH	AnterSeptal	−15.95 ± 5.19	−25.80 ± 4.59	< 0.001	−11.32 ± 4.23	−21.05 ± 3.45	< 0.001	−8.50 ± 3.70	−17.55 ± 2.77	< 0.001
	Posterior	−16.96 ± 5.13	−24.18 ± 3.97	< 0.001	−13.43 ± 4.35	−20.40 ± 3.67	< 0.001	−10.94 ± 3.77	−17.50 ± 3.61	< 0.001
4-CH	Lateral	−17.19 ± 7.11	−24.33 ± 3.77	< 0.001	−13.08 ± 6.11	−20.32 ± 3.39	< 0.001	−10.20 ± 5.37	−17.32 ± 3.24	< 0.001
	Septal	−16.70 ± 5.41	−24.11 ± 3.61	< 0.001	−13.73 ± 4.71	−20.75 ± 3.15	< 0.001	−11.91 ± 4.24	−18.30 ± 2.85	< 0.001
2-CH	Anterior	−15.30 ± 6.69	−23.77 ± 3.46	< 0.001	−11.10 ± 5.89	−20.31 ± 2.89	< 0.001	−8.36 ± 5.20	−17.80 ± 2.55	< 0.001
	Inferior	−17.74 ± 5.21	−25.40 ± 4.24	< 0.001	−14.50 ± 4.30	−21.96 ± 3.61	< 0.001	−12.58 ± 3.88	−19.41 ± 3.11	< 0.001
Global		−16.75 ± 4.13	−23.99 ± 3.05	< 0.001	−13.44 ± 3.68	−20.64 ± 2.59	< 0.001	−10.85 ± 3.28	−17.82 ± 2.28	< 0.001

### Segmental longitudinal rotation

**Fig. 2:**
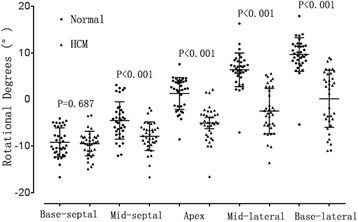
Scatter diagram was used to directly reflect the peak segmental longitudinal rotational degrees in the systolic period between normal subjects and HCM patients

The lateral wall rotated counter-clockwise, whereas the septum wall rotated clockwise in the normal subjects, the rotational degree was similar, whereas the direction was opposite (Table 3, Fig. 2). When the segmental longitudinal rotation of HCM patients and normal subjects were compared, the rotational degree of the midmyocardial-septal, apex, and the lateral wall of HCM patients was significantly different relative to that of the normal subjects. The rotational motion of the LV septal, apex, and lateral walls in HCM patients were impaired.

**Table 3:** Comparison of the peak segmental and global longitudinal rotational degrees in the systolic period between HCM patients and normal subjects (mean ± s.d.)

	Base-Septal(°)	Mid-Septal(°)	Apex(°)	Mid-lateral(°)	Base-lateral(°)	Global
HCM (36)	−9.45 ± 2.65	−7.90 ± 3.08	−5.07 ± 3.61	−2.49 ± 4.85	0.15 ± 6.14	−4.92 ± 2.65
Normal (36)	−9.21 ± 3.11	−4.52 ± 4.01	1.28 ± 3.42	6.38 ± 3.63	9.66 ± 3.63	0.02 ± 2.42
*P*-Value	0.687	< 0.001	< 0.001	< 0.001	< 0.001	< 0.001

*Base-Septal* the base of the septal wall, *Mid-Septal* the middle of the septal wall, *Apex* the apex of the left ventricular, *Mid-lateral* the middle of the lateral wall, Base-lateral: the base of the lateral wall

### Globe longitudinal rotation of LV curves in the cardiac

**Fig. 3:**
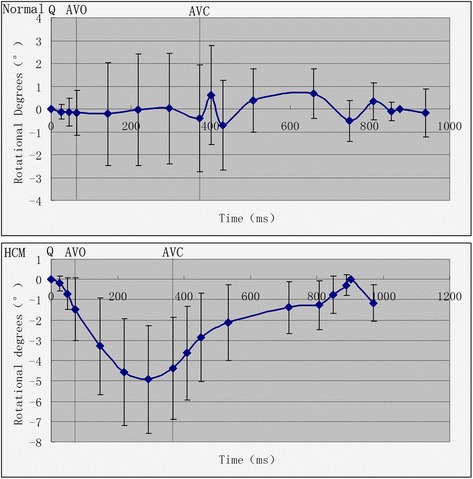
The curve of the longitudinal rotational degrees in normal subjects and HCM patients at 17 different points in two cardiac cycles

The longitudinal rotation degrees in normal subjects was <3°, around the zero baseline for a small angle movement. In HCM patients, the clockwise longitudinal rotation was found (Table 4, Fig. 3).

**Table 4:** Longitudinal rotational degrees in HCM patients and normal subjects at 17 different points in two cardiac cycles (mean ± s.d.)

	HCM (36)		Normal (36)	
Points	Time(ms)	Rotation Degree(°)	Time(ms)	Rotation Degree(°)
Q	0	0	0	0
MVC	25 ± 7	−0.19 ± 0.36	25 ± 6	−0.12 ± 0.32
IVS	48 ± 8	−0.70 ± 0.79	45 ± 7	−0.13 ± 0.62
AVO	73 ± 13	−1.47 ± 1.55	65 ± 13	−0.17 ± 0.98
25%	147 ± 15	−3.29 ± 2.40	142 ± 15	−0.22 ± 2.24
50%	220 ± 21	−4.57 ± 2.63	219 ± 18	−0.02 ± 2.44
75%	293 ± 29	−4.92 ± 2.65	296 ± 23	0.02 ± 2.42
AVC	367 ± 37	−4.38 ± 2.53	373 ± 28	−0.41 ± 2.35
IVR	409 ± 35	−3.63 ± 2.30	402 ± 30	0.61 ± 2.17
MVO	451 ± 43	−2.86 ± 2.17	431 ± 37	−0.71 ± 1.97
E-Peak	534 ± 53	−2.12 ± 1.86	507 ± 41	0.36 ± 1.39
E-End	716 ± 95	−1.38 ± 1.29	658 ± 70	0.68 ± 1.09
A-Onset	808 ± 190	−1.26 ± 1.21	749 ± 104	−0.52 ± 0.90
A-Peak	848 ± 128	−0.74 ± 0.92	809 ± 104	0.34 ± 0.80
A-End	890 ± 141	−0.28 ± 0.53	854 ± 105	−0.11 ± 0.41
Q-2	904 ± 147	0	875 ± 98	0
AVO-2	971 ± 152	−1.16 ± 0.89	940 ± 103	−0.18 ± 1.05

When viewed from the above values, positive values of the rotation degree were considered as count-clockwise rotation, while negative values were considered as clockwise rotation

*MVC* mitral valve closure, *IVS* isovolumic contraction, *AVO* aortic valve opening, *AVC* aortic valve closure, *IVR* isovolumic relaxation, *MVO* mitral valve opening

### Correlation between IVSD and the peak longitudinal systolic strain in myocardial layers

**Fig. 4:**
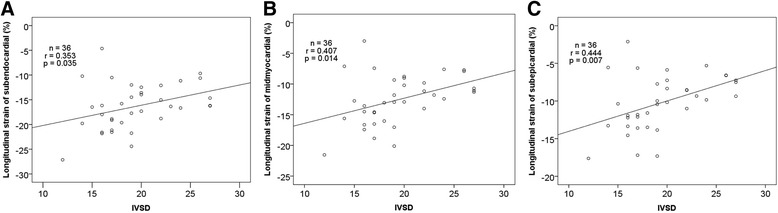
The correlation between IVSD and the longitudinal strain of the subendocardial (**a**), midmyocardial (**b**) and subepicardial layers (**c**) in HCM patients

The IVSD positively correlated with the peak longitudinal systolic strain of the subendocardial, midmyocardial, and subepicardial layers in the HCM patients (subendocardial, *r* = 0.353, *P* = 0.035; midmyocardial, *r* = 0.407, *P* = 0.014; subepicardial, *r* = 0.444, *P* = 0.007; (Table 5, Fig. 4). Therefore, patients with higher IVSD had higher peak longitudinal systolic strain in the different myocardial layers.

**Table 5:** Correlation between IVSD and the longitudinal strain of the subendocardial, midmyocardial and subepicardial layers in HCM patients

	Subendocardial	Midmyocardial	Subepicardial
r-value	0.353	0.407	0.444
*p*-value	0.035	0.014	0.007

### ROC analysis for detecting the accuracy of the different layers of the peak longitudinal systolic strain in HCM patients

**Fig. 5:**
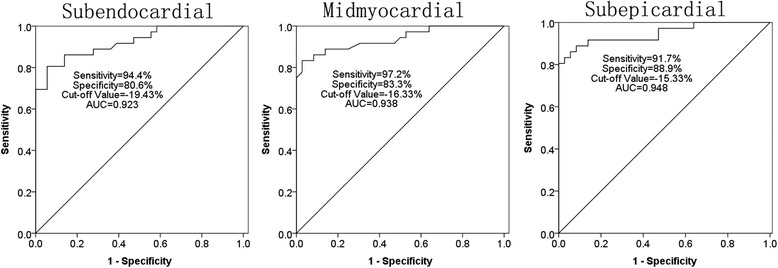
ROC analysis for detecting the accuracy of the peak longitudinal systolic strain of different myocardial layers in HCM patients. The area under ROC curve values for the subendocardial, midmyocardial and subepicardial layers were 0.923, 0.938, 0.948, respectively. Sensitivity for the subendocardial, midmyocardial and subepicardial layers were 94.4%, 97.2% and 91.7%, respectively. Specificity for the subendocardial, midmyocardial and subepicardial layers were 80.6%, 83.3% and 88.9%, respectively. Cut-off values for the subendocardial, midmyocardial and subepicardial layers were −19.43%, −16.33% and −15.33%, respectively

Area under ROC curves allowing determination of optimal cut-off values for sensitivity, specificity, and accuracy for the peak longitudinal systolic strain of different layers in assessing the LV function. The area under ROC curve values for subendocardial, midmyocardial and subepicardial layers in HCM patients were 0.923, 0.938, 0.948. The sensitivity was higher for peak longitudinal systolic strain of midmyocardial layer (97.2%) than for the subendocardial and subepicardial layers (94.4% and 91.7%). Specificity was higher for peak longitudinal systolic strain of subepicardial layer (88.9%) than for the subendocardial and midmyocardial layers (80.6% and 83.3%). The cut-off values of the subendocardial, midmyocaidial and subepicardial were −19.43%, −16.33% and −15.33%. (Fig. 5).

#### Reproducibility and repeatability

Interobserver measurement of the global strain and LR were determined by having a second investigator measure all chosen subjects. For intraobserver variability, all subjects were analyzed twice by one investigator, and the second intraobserver measurements were “blinded” to results from the initial measurements. The results for the intraobserver and interobserver variabilities for the peak systolic global strain of subendocardial, midmyocardial and subepicardial layers and longitudinal rotation degrees upon repeated measurements in all study patients were shown in Table 6.

**Table 6:** Interobserver and intraobserver reproducibility and repeatability

		Interobserver			Intraobserver		
		HCM	Normal	*p*-value	HCM	Normal	*p*-value
Global Strain (%)	subendocardial	−16.94 ± 4.47	−24.08 ± 3.41	< 0.001	−17.63 ± 5.20	−23.88 ± 3.72	< 0.001
	midmyocardial	−13.60 ± 3.90	−21.01 ± 2.99	< 0.001	−14.39 ± 4.56	−20.60 ± 3.34	< 0.001
	subepicardial	−10.98 ± 3.48	−18.43 ± 2.70	< 0.001	−11.76 ± 4.02	−17.86 ± 3.12	< 0.001
Global LR(°)		−4.97 ± 2.66	−0.05 ± 2.44	< 0.001	−4.88 ± 2.59	−0.03 ± 2.39	< 0.001

## Discussion

This study investigated the differences in LV longitudinal systolic function of HCM patients relative to healthy subjects, with 4 main findings. First, in HCM patients decreased regional LV peak systolic longitudinal strain appeared not only in hypertrophied LV myocardium, but also in non-hypertrophied myocardium. Clockwise longitudinal rotation was found in the HCM patients, and the interventricular septum thickness at end-diastole positively correlated with the peak longitudinal systolic strain of the different layers. Finally, the area under the ROC curve values for the subendocardial, midmyocardial and subepicardial layers were 0.923, 0.938, 0.948, respectively. The sensitivity was higher for peak longitudinal systolic strain of the midmyocardial layer (97.2%) than for the subendocardial and subepicardial layers (94.4% and 91.7%). Specificity was higher for the peak longitudinal systolic strain of the subepicardial layer (88.9%) than for the subendocardial and midmyocardial layers (80.6% and 83.3%). Cut-off values for the subendocardial, midmyocardial and subepicardial layers were −19.43%, −16.33% and −15.33%.

HCM is a very common and important cardiac disease. LV hypertrophy, myocardial fibrosis, and fiber disarray in the LV myocardium has been reported as the major structural myocardial abnormalities in HCM patients [4], and systolic function is damaged thereby. LV hypertrophy is the result of compensatory myocardial function.

Systolic function by conventional measurements, such as LVEF, cannot detect cardiac myocardium impairment; microscopic abnormalities result in intrinsic functional abnormalities [24]. Cardiac function has been determined based on velocity, strain, strain rate, degrees of rotation, and torsion using 2D–STE in many heart diseases, including cardiomyopathy, coronary heart disease, and hypertension [8, 25–28]. However, to measure the peak systolic strain of the subendocardial, midmyocardial, and subepicardial layers is a novel method for adjudging myocardium function. One of the innovations of the present study was to use 2D–STE to measure the peak systolic longitudinal strain of the subendocardial, midmyocardial, and subepicardial layers in HCM patients, and then evaluate longitudinal systolic function in HCM patients relative to that of healthy individuals.

In the present study, the peak systolic longitudinal strain of the subendocardial, midmyocardial, and subepicardial layers in both HCM and healthy individuals was: subendocardial > middle myocardial > subepicardial. A normal myocardium consists of the subendocardium, middle, and subepicardium fibers. Longitudinally oriented fibers of the subendocardium and subepicardium lead to longitudinal contraction, and middle wall fibers that are circumferentially oriented lead to circumferential shortening. Differences in contraction of the subepicardial and subendocardial layers lead to high subendocardial strain. The subendocardial region is responsible for most of the longitudinal deformation.

The present result was consistent with previous studies [4, 21]. By detecting the peak systolic longitudinal strain of the subendocardial, midmyocardial, and subepicardial layers, our data showed attenuation of longitudinal systolic function of the LV myocardium in HCM patients. Popovic et al. [29] also demonstrated that myocardial fibrotic lesions in the LV myocardium were associated with reduced longitudinal strain in HCM patients, and fibrotic lesions and wall thickening were predictors of lower longitudinal strain. Kofflard et al. [30] considered that the decrease in coronary flow reserve in HCM patients predisposed to myocardial ischemia. According to the research, they found that in HCM patients, hemodynamic (LV end-diastolic pressure, LV outflow tract gradient), echocardiographic (indexed LV mass) and histological (% luminal area of the arterioles) changes are responsible for a decrease in coronary flow reserve. Because of these changes, the systolic function in HCM patients was impaired. From our present results, we conclude that longitudinal function was damaged in HCM patients, and longitudinal strain of the different myocardial layers can sensitively reflect cardiac systolic function.

The different orientation of the ventricular muscle fibers led to the different motion of the heart. In the short-axis view, when viewed from the apex, the LV apex rotates counterclockwise, whereas the base rotates clockwise in systole period. However, when a normal heart is viewed from the long-axis view, the motion can be described as shortening of its long axis and thickening of its walls [31, 32].

Longitudinal rotation, first discussed by Popovic et al. [31], refers to rotational motion in the longitudinal direction. Some researchers [28, 32] have found longitudinal rotation in patients with dilated cardiomyopathy, primary hypertension, and other heart diseases. In the present study, clockwise longitudinal rotation was found in HCM patents. The curves of normal subjects showed longitudinal rotations <3°, around the zero baseline for a small angle movement. However, in HCM patients, clockwise longitudinal rotation was found in the heart. The segmental rotation motion in HCM patients also differed from that of the healthy control subjects. In the normal subjects, the lateral wall rotated counter-clockwise, whereas the septum wall rotated clockwise, the rotation degrees were similar, but the direction was the opposite.

In the HCM patients of the present study, the rotational motion of the septum, apex, and the lateral wall of LV also differed from that of the controls. Differences in the segmental and global longitudinal rotation were associated with the unique distribution of myocardium disarray in the HCM patients. The myocardial hypertrophy and fibrosis of these patients was probably responsible for the global and regional abnormalities of the LV myocardial mechanics. When the heart contracted, the abnormal balance of the various myocardial layers resulted in aberrant differences in rotational degrees and the direction of global longitudinal rotation. We also considered that neural and humoral regulation mechanisms may underlie the orientation of the longitudinal rotation. Further researches are necessary to confirm this hypothesis.

In the present study, the IVSD of the HCM patients was found to correlate positively with the peak longitudinal systolic strain of the subendocardial, midmyocardial, and subepicardial layers; thickening of the IVSD in HCM patients was consistent with its systolic function. We therefore conclude that obvious thickening of the IVSD reflects impaired longitudinal systolic function.

The ROC analysis for detecting the accuracy of the peak longitudinal systolic strain showed that the area under ROC curve values for subendocardial, midmyocardial and subepicardial layers were 0.923, 0.938, 0.948. The sensitivity was higher for peak longitudinal systolic strain of midmyocardial layer (97.2%) than for the subendocardial and subepicardial layers (94.4% and 91.7%). Specificity was higher for peak longitudinal systolic strain of subepicardial layer (88.9%) than for the subendocardial and midmyocardial layers (80.6% and 83.3%). From ROC analysis, we knew that using 2D–STE for detecting the peak longitudinal systolic strain of HCM is accurately. The results also showed that the LV function was impaired in HCM patients.

## Conclusion

Longitudinal function in HCM is damaged, despite normal LVEF. The changes of peak systolic longitudinal strain of the subendocardial, midmyocardial, and subepicardial layers, and the longitudinal rotation detected by 2D–STE can reflect the LV systolic dysfunction in HCM patients. In clinician, early detection of LV dysfunction in HCM patients can make us to understand the pathophysiology of HCM better, and it also can help the physician to have an earlier symptomatic treatment and then compare the efficacy of the different drugs. The peak longitudinal systolic strain of the subendocardial, midmyocardial, and subepicardial layers and the longitudinal rotation (detected by 2D–STE) are very sensitive determinants of systolic function in patients with HCM.

### Limitation

The high standard deviations are indicative of the high variability with this technique - when narrow ROI is chosen to try and assess myocardial layers, higher strain values (more deformation) are recorded and more noise is introduced.

## Abbreviations

2D–STE: 2-dimensional speckle tracking echocardiography
HCM: Hypertrophic cardiomyopathy
IVSD: Interventricular septal thickness in end-diastolic period
LAD: Left atrial diameter
LV: Left ventricular
LVEDV: Left ventricular end-diastolic volume
LVEF: Left ventricular ejection fraction
LVESV: Left ventricular end-systolic volume
LVH: Left ventricular hypertrophy
LVPWD: LV posterior wall thickness in end-diastolic period
